# Immunocytes Play a Crucial Role as Mediators in the Protective Effects of D-β-Hydroxybutyrate Dehydrogenase 1 against Type 2 Diabetes Mellitus: A Mendelian Randomization Study

**DOI:** 10.2174/0118715303380282250225071730

**Published:** 2025-05-19

**Authors:** Yi-Ying Liu, Yue-Yang Zhang, Qin Wan

**Affiliations:** 1 Centre for Endocrine and Thyroid Diseases, Deyang People's Hospital, Deyang, 618000, China;; 2 Department of Endocrinology and Metabolism, Affiliated Hospital of Southwest Medical University, Luzhou, 646000, China;; 3 Metabolic Vascular Disease Key Laboratory of Sichuan Province, Luzhou, 646000, China;; 4 Sichuan Clinical Research Center for Diabetes and Metabolism, Luzhou, 646000, China;; 5 Sichuan Clinical Research Center for Nephropathy, Luzhou, China, 646000;; 6 Cardiovascular and Metabolic Diseases Key Laboratory of Luzhou, Luzhou, 646000, China

**Keywords:** D-β-hydroxybutyrate dehydrogenase, immunocyte, type 2 diabetes, mendelian randomization, mediation, randomization (MR) analysis

## Abstract

**Background:**

Observational studies suggest an association between the immune system and type 2 diabetes. The present study sought to ascertain the causal relationship between BDH1 and type 2 diabetes and investigate whether immunocytes mediate this relationship.

**Methods:**

Appropriate single nucleotide polymorphisms (SNPs) were carefully selected from publicly available GWAS databases based on rigorous criteria to ensure the validity of the Mendelian randomization (MR) analysis. Inverse variance weighting (IVW) was employed as the primary approach for assessing effect sizes, supplemented by four sensitivity analysis techniques: weighted median, simple mode, weighted mode, and MR-Egger regression tests, all aimed at ensuring the robustness and reliability of the IVW results. Reverse MR was conducted to confirm the feasibility of the mediation analysis. Lastly, Cochran’s Q test, MR Egger intercept regression, and MR-PRESSO analysis were utilized to examine heterogeneity and horizontal pleiotropy.

**Results:**

The expression of BDH1 is inversely associated with the risk of type 2 diabetes, with an odds ratio of 0.97 (95% CI: 0.95-0.99). IgD^+^ CD38^+^ B cell absolute count (20.7%), HLA DR on dendritic cell (18.7%), BAFF-R on CD20- CD38- B cell (9.5%), CD25 on IgD^+^ CD24^+^ B cell (4.1%), and BAFF-R on IgD^+^ B cell (3.4%), all exhibit certain mediating effects, whereas IgD^+^ CD38^+^ B cell absolute count, activated and resting CD4 regulatory T cell %, CD4^+^ T cell, transitional B cell absolute count, CD28- CD8 dim T cell absolute count, CD45 on HLA DR^+^ CD8^+^ T cell, FSC-A on HLA DR^+^ natural killer, and SSC-A on plasmacytoid dendritic cell exert masking effects.

**Conclusion:**

The findings indicate that immunocytes could serve as a crucial mediating mechanism through which BDH1 exerts its protective effect against type 2 diabetes, offering novel insights for the prevention and therapeutic management of the disease.

## 1 INTRODUCTION

Type 2 diabetes stands as one of the most prevalent and consequential diseases within the endocrine system [1]. It is characterized by insulin resistance accompanied by relative insulin deficiency, often evidenced by markedly elevated fasting blood sugar levels [2, 3]. According to the International Diabetes Federation (IDF) report of 2021, an estimated 537 million adults aged 20 to 79 worldwide are afflicted with diabetes, representing one in every ten adults. Projections indicate that by 2030, this number will escalate to 643 million, and by 2045, it will reach 783 million [4]. Such a substantial trajectory underscores the increasingly burdensome impact of diabetes [5-7]. The World Health Organization (WHO) has further reported that in 2019 alone, over 150 million individuals succumbed to diabetes, rendering it the ninth leading cause of mortality globally [8, 9].

Type 2 diabetes is commonly recognized as a complex disorder influenced by a myriad of risk factors, including smoking, unhealthy diet, advanced age, obesity, psychological stress, cardiovascular conditions, and genetic predispositions [10-13]. In efforts to elucidate the underlying mechanisms of type 2 diabetes, numerous researchers have expanded their investigations into alternative avenues. Presently, the concept of “immuno-metabolism” has emerged as a focal point of research interest. While immune system dysregulation has traditionally been associated with type 1 diabetes, extensive genetic, epidemiological, and physiological studies have unveiled the significant involvement of the immune system in the pathogenesis of type 2 diabetes [14-16]. Lu *et al*. [17] revealed that inflammasomes can directly or indirectly modulate insulin signaling pathways, thereby contributing to insulin resistance and the onset of type 2 diabetes.

β-hydroxybutyrate dehydrogenase 1 (BDH1) serves as a pivotal rate-limiting enzyme in ketone metabolism, directly facilitating the breakdown of β-hydroxybutyrate. Previous studies have elucidated the inhibitory effect of the mitochondrial β-oxidation enzyme HADHA on glucagon response in the liver and pancreas by promoting β-hydroxybutyrate production [18]. Ketone bodies, recognized as vital alternative energy substrates under stress conditions, are increasingly regarded as promising therapeutic targets for a wide range of diseases, including cancer [19]. BDH1 has been demonstrated to play a pivotal role in determining the fate of adipocytes and has been shown to exert anti-tumor effects in acute leukemia [20]. Furthermore, the upregulation of Bdh1 expression mitigates oxidative stress and inflammation, contributing to the amelioration of atherosclerosis [21]. Our laboratory investigations have demonstrated that BDH1 may ameliorate diabetic nephropathy and atherosclerosis associated with type 2 diabetes by modulating NRF2 [22]. However, the existing literature is limited in its examination of the interplay among BDH1, immunocytes, and type 2 diabetes.

Mendelian randomization (MR) stands as a recognized statistical method utilized to assess causality between exposures and diseases, employing genetic variation as an instrumental variable for statistical analysis [23]. Grounded in Mendel's law of inheritance, genetic variations are randomly assorted at conception and precede the onset of any disease, thereby minimizing the impact of confounding factors and reverse causality [24]. Therefore, drawing upon existing studies, we hypothesize that the upregulation of BDH1 expression may confer a protective effect against type 2 diabetes, with immune cells playing a pivotal role in mediating this process. Through these endeavors, we aim to contribute to the development of more rational and productive prevention and treatment strategies for type 2 diabetes.

## 2 METHODS

### 2.1 Study Design

This study aimed to assess the causal relationship between BDH1 and type 2 diabetes and investigate the potential mediating role of immunocytes through bidirectional two-sample MR. All MR analyses are predicated on three key assumptions. Firstly, genetic variation must be closely associated with the exposure of interest. Secondly, genetic variation should remain unaffected by both known and unknown confounding factors. Lastly, genetic factors should solely influence outcome factors through the exposure of interest [25, 26].

### 2.2 Data Sources

The eQTL data for BDH1 were obtained from summary-level data derived from the first-stage study, which comprised 31,684 individuals of European ancestry and was conducted by the eQTLGen Consortium. Only cis-eQTLs were utilized as genetic instruments [27]. The immunocyte phenotypes were sourced from a GWAS analysis involving 3,757 Sardinian individuals, encompassing a total of 731 immunocyte phenotypes [28]. The outcome data for type 2 diabetes were obtained from meta-analyses of multiple GWAS studies, which included 38,841 type 2 diabetes patients and 451,248 European ancestry controls [29].

### 2.3 Instrument Selection

Firstly, to ensure adequate single nucleotide polymorphisms (SNP) for statistical analysis, we applied significance cutoff thresholds of 5×10^-8^ and 1×10^-5^ for BDH1 and immunocytes, respectively [30]. We then removed linkage disequilibrium (R^2^ < 0.001 within a 10,000-kb distance) to obtain independent genetic variants. Additionally, to mitigate bias arising from weak instrument variables (IVs), we computed the F statistics for all SNPs utilized in the analysis, retaining those with F > 10 as strong instrument variables. Subsequently, SNPs meeting the requisite assumptions were extracted from outcome data and merged with exposure SNPs. Palindrome sequences were excluded, and the resulting SNPs were utilized for subsequent statistical analysis.

### 2.4 Statistical Analysis

We applied five common MR analysis methods: inverse variance weighted (IVW), weighted median, simple mode, weighted mode, and MR-Egger regression test. Given that IVW demonstrates superior statistical efficacy in the absence of horizontal pleiotropy [31], IVW was designated as the primary analysis method, with the remaining four methods serving as auxiliary approaches. The results from the four sensitivity analyses were deemed to bolster the reliability of the IVW findings, provided they were directionally consistent with the IVW outcomes [32]. Our analysis proceeded in several sequential steps. Firstly, we estimated the causal relationship between BDH1 and type 2 diabetes, complemented by reverse MR analysis to examine potential reverse causality between them. Next, we assessed the causal relationship between BDH1 and immunocyte phenotypes, followed by an examination of the causal relationship between immunocyte phenotypes and type 2 diabetes. Subsequently, Cochran's Q test was employed to evaluate heterogeneity (*p* < 0.05), while the MR-Egger intercept test was utilized to assess the presence of horizontal pleiotropy (*p* < 0.05). MR- PRESSO was subsequently deployed to comprehensively evaluate SNPs exhibiting horizontal pleiotropy, identify and address outliers as necessary, and furnish corrected results [33]. Finally, we decomposed the total effect of BDH1 on type 2 diabetes into direct and indirect effects. The coefficient product method facilitated the estimation of the indirect effect, representing the influence of BDH1 on type 2 diabetes through mediation. The proportion of mediation corresponding to each mediator was determined by dividing the indirect effect by the total effect [34]. When the indirect effect contrasts with the total effect, immune cells are considered to exert masking effects.

All analyses were conducted using the TwoSampleMR package (version 0.5.6) in R software (version 4.3.3). A two-tailed *p*-value of less than 0.05 was deemed statistically significant.

## 3 RESULTS

### 3.1 Instrument Selection

After rigorous screening, we identified all IVs that met the criteria for subsequent analyses. In both the forward Mendelian randomization (MR) investigation of BDH1 and type 2 diabetes and the reverse MR analysis, all SNPs exhibited F statistics exceeding 10. Notably, weak instrument bias did not significantly impact the results, as detailed in Tables (**S1-S3**).

### 3.2 Causal Relationship between BDH1 and Type 2 Diabetes Mellitus

Fig. (**1**) and Table **S4** present the results of the IVW analysis, indicating the expression of the BDH1 gene to be associated with a reduced risk of type 2 diabetes mellitus, with an odds ratio of 0.97 (95% CI: 0.95-0.99, *P* = 3.72E-03). The findings from the four additional sensitivity analyses were consistent with the IVW analysis. Notably, Cochran’s Q test revealed no evidence of heterogeneity in the results (*P* > 0.05), and both the MR-Egger intercept and the global test suggested no presence of horizontal pleiotropy (*P* > 0.05). Furthermore, the reverse MR analysis indicated no reverse causal relationship between the BDH1 gene and type 2 diabetes mellitus, with an odds ratio of 0.97 (95% CI: 0.92, 1.02, *P* > 0.05), thereby meeting the prerequisites for conducting mediation analysis.

### 3.3 Causal Relationship between BDH1 and Immunocyte Phenotype

Fig. (**1**) and Table **S5** illustrate the causal relationship between BDH1 and 24 immunocyte phenotypes. Notably, 12 of these phenotypes exhibited negative correlations, including IgD^+^ CD38^+^ B cell absolute count (OR: 0.90, 95% CI: 0.80-0.97; *P* = 4.25E-03), CD86^+^ myeloid dendritic cell absolute count (0.89, 0.81-0.96, *P* = 4.10E-03), activated and resting CD4 regulatory T cell % and CD4^+^ T cell (0.89, 0.80-0.99, *P* = 0.03), naive CD4^+^ T cell absolute count (0.86, 0.80-0.93, *P* = 6.49E-05), transitional B cell absolute count (0.87, 0.81-0.94, *P* = 4.45E-04), CD28- CD8 dim T cell absolute count (0.89, 0.82-0.97, *P* = 5.03E-03), BAFF-R on CD20- CD38- B cell (0.86, 0.80-0.93, *P* = 1.94E-04), BAFF-R on IgD^+^ B cell (0.92, 0.85-1.00, *P* = 0.04), CD25 on IgD^+^ CD24^+^ B cell (0.91, 0.83-0.99, *P* = 0.03), FSC-A on HLA DR^+^ natural killer (0.90, 0.83-0.98, *P* = 0.02), SSC-A on plasmacytoid dendritic cell (0.83, 0.77-0.91, *P* = 3.28E-05), HLA DR on plasmacytoid dendritic cell (0.81, 0.75-0.88, P = 1.03E-06), and HLA DR on dendritic cell (0.85, 0.78-0.92, *P* = 1.44E-04). Conversely, positive correlations were observed in certain phenotypes, such as CD45 on HLA DR^+^ CD8^^+^^ T cell (1.13, 1.04-1.23, *P* = 3.47E-03) and CD45 on CD33- HLA DR^+^ (1.18, 1.05-1.32, P = 4.03E-03). Moreover, as depicted in Table **S6**, MR-Egger intercept analysis revealed the absence of statistically significant horizontal pleiotropy across the analyses. Additionally, Cochran's Q test identified heterogeneity solely in activated and resting CD4 regulatory T cell % and CD4^+^ T cells. 

### 3.4 Causal Relationship between Immunocyte Phenotype and Type 2 Diabetes Mellitus

Fig. (**1**) and Tables **S7-S8** demonstrate causal associations between 39 immunocyte phenotypes and type 2 diabetes mellitus. Notably, IgD^+^ CD38^+^ B cell absolute count (0.99, 0.98-1.00, *P* = 0.03), activated and resting CD4 regulatory T cell % and CD4^+^ T cell (0.97, 0.95-0.99, *P* = 0.01), naive CD4^+^ T cell absolute count (1.05, 1.01-1.08, *P* = 8.45E-03), transitional B cell absolute count (0.98, 0.96-0.99, *P* = 1.93E-03), CD28- CD8 dim T cell absolute count (0.98, 0.96-0.99, *P* = 8.24E-03), BAFF-R on CD20- CD38- B cell (1.02, 1.00-1.14, *P* = 0.02), BAFF-R on IgD^+^ B cell (1.01, 1.00-1.03, *P* = 0.04), CD25 on IgD^+^ CD24^+^ B cell (1.01, 1.00-1.03, *P* = 0.01), CD45 on HLA DR^+^ CD8^^+^^ T cell (1.02, 1.00, 1.04-*P* = 0.03), FSC-A on HLA DR^+^ natural killer cell (0.98, 0.97-0.99, *P* = 3.49E-03), SSC-A on plasmacytoid dendritic cell (0.98, 0.96-0.99, *P* = 9.87E-03), and HLA DR on dendritic cell (1.04, 1.02-1.06, *P* =3.12E-04) exhibited significant causal links with type 2 diabetes mellitus. As presented in Table **S8**, sensitivity analysis results indicated acceptable heterogeneity for CD62L- plasmacytoid dendritic cell absolute count, activated and resting CD4 regulatory T cell % and CD4^^+^^ T cell, naive CD4^^+^^ T cell absolute count, CD19 on IgD- CD24- B cell, CD20 on IgD^+^ CD38- naive B cell, CD45 on HLA DR^+^ CD8^^+^^ T cell, CD127 on CD45RA^+^ CD4^^+^^ T cell, HLA DR on myeloid dendritic cell, HLA DR on plasmacytoid dendritic cell, HLA DR on dendritic cell, and HLA DR on CD33- HLA DR^+^, when using IVW analysis. Furthermore, MR-Egger intercept analysis confirmed the absence of horizontal pleiotropy across all results, except for CD86^+^ myeloid dendritic cell absolute count, CD62L- plasmacytoid dendritic cell absolute count, and HLA DR on plasmacytoid dendritic cell.

### 3.5 Mediation Analysis

Fig. (**2**) and Table **S9** illustrate the contributions of 12 immunocyte phenotypes to the protective effect of BDH1 on type 2 diabetes mellitus. Among them, naive CD4^+^ T cell absolute count accounted for 20.7% (0%, 53.5%) of the total effect, while HLA DR on dendritic cell accounted for 18.7% (0%, 61.9%), BAFF-R on CD20- CD38- B cell accounted for 9.5% (0%, 43.8%), CD25 on IgD^+^ CD24^+^ B cell accounted for 4.1% (0%, 28.6%), and BAFF-R on IgD^+^ B cell accounted for 3.4% (0%, 22.5%). However, due to masking effects exerted by IgD^+^ CD38^+^ B cell absolute count, activated and resting CD4 regulatory T cell % and CD4^+^ T cell, transitional B cell absolute count, CD28- CD8 dim T cell absolute count, CD45 on HLA DR^+^ CD8^+^ T cell, FSC-A on HLA DR^+^ natural killer, and SSC-A on plasmacytoid dendritic cell, the mediation proportion could not be calculated.

## 4 DISCUSSION

In our MR analysis, we observed evidence supporting a causal protective effect of BDH1 on type 2 diabetes, with the involvement of 7 immunocyte phenotypes in this protective mechanism. Specifically, IgD^+^ CD38^+^ B cell absolute count (20.7%), HLA DR on dendritic cell (18.7%), BAFF-R on CD20- CD38- B cell (9.5%), CD25 on IgD^+^ CD24^+^ B cell (4.1%), and BAFF-R on IgD^+^ B cell (3.4%) exhibited mediating effects. However, IgD^+^ CD38^+^ B cell absolute count, activated and resting CD4 regulatory T cell % and CD4^+^ T cell, transitional B cell absolute count, CD28- CD8 dim T cell absolute count, CD45 on HLA DR^+^ CD8^+^ T cell, FSC-A on HLA DR^+^ natural killer, and SSC-A on plasmacytoid dendritic cell showed masking effects.

BDH1, an enzyme facilitating the interconversion of acetoacetate and β-hydroxybutyrate coupled with NAD^+^/NADH, plays pivotal roles in cellular metabolism, lipid metabolism, and redox reactions [35, 36]. Numerous studies have established close links between BDH1 and conditions, such as pregnancy and cardiovascular diseases [37, 38]. Brahma *et al*. [39] revealed that diabetes can hinder heart ketone oxidation by regulating gene expression and promoting heart metabolic reprogramming, leading to diminished expression of heart BDH1 and succinyl-CoA transferase. This finding further underscores the protective role of BDH1 in type 2 diabetes.

While no direct studies currently link BDH1 to the immune system, the ketone body metabolism in which BDH1 participates is thought to be closely intertwined with immune system functions [40]. On one hand, ketone bodies directly influence immune cells, activating pathways, such as AMPK, and promoting anti-inflammatory responses [41]. On the other hand, a study by Luda, *et al*. [42] found that CD8^+^ T effector cells preferentially utilize ketone bodies for energy over glucose, and ketone bodies can modulate T cell function by influencing histone acetylation. Notably, the study also revealed that the impact of βOHB on the bioenergetics of CD8^+^ T cells is contingent upon the expression of BDH1. This evidence offers further theoretical support for this study. Additionally, some studies suggest that CD8^+^ T montent>ells transfer acetyl-CoA to synthesize βOHB, thereby ensuring the survival of CD8^+^ T mtent> cells through histone β-hydroxybutyrylation [43].

While extensive research has delved into the pathogenesis of type 2 diabetes, achieving a consensus remains elusive due to the multifactorial nature of the disease [44]. In recent years, mounting evidence has highlighted the intricate interplay between type 2 diabetes and the immune system [45].

 Stentz [46] suggested that type 2 diabetes correlates with the overactivation of CD4^+^ T lymphocytes. Specifically, CD4^+^ T lymphocytes tend to differentiate into pro-inflammatory Th1 and Th17 cells in individuals with type 2 diabetes [47]. Moreover, research has revealed a reduction in the expression of Nrf2 and its downstream antioxidant-related substances in type 2 diabetes patients, with circulating Nrf2 levels negatively correlated with Th1 levels [48]. Additionally, CD8^^+^^ T lymphocytes have been implicated in the synthesis and expression of the pro-inflammatory factor IL-17, with their accumulation potentially triggering tissue inflammation and insulin resistance [49].

The impairment of natural killer (NK) cells' function in patients with type 2 diabetes has been observed, characterized by reduced degranulation capacity [50]. Additionally, observational studies have highlighted a significant negative correlation between circulating NK cell levels and HbA1c levels [51]. Furthermore, macrophages within adipose tissue play a crucial role in regulating adipose tissue inflammation. Elevated levels of interleukin-6 (IL-6) have been linked to insulin resistance and the progression of type 2 diabetes. Moreover, increased expression of nuclear lamin A/C in type 2 diabetes patients further exacerbates the expression of corresponding inflammatory factors [52]. While observational studies have underscored the intricate relationship between the immune system and type 2 diabetes development, they fall short of establishing causality. Li [53] provided compelling evidence linking higher levels of monocytes and T lymphocyte subpopulations in circulation to increased susceptibility to type 2 diabetes, consistent with our research findings.

### 4.1 Strengths and Limitations

Our study boasts several strengths, primarily leveraging large-scale GWAS data, ensuring robustness through substantial sample sizes and high statistical power. By utilizing SNPs with F-statistics exceeding 10, we mitigated biases associated with weak instrumental variables. Furthermore, comprehensive sensitivity analyses bolstered the reliability of our findings.

Nevertheless, certain limitations warrant consideration. Firstly, due to our inability to filter the necessary genetic information from available GWAS studies conducted on non-European populations, our reliance on GWAS data predominantly sourced from European populations necessitates caution when extrapolating findings to other demographic groups. Additionally, the inherent constraints of exploring multiple exposures using two-sample MR may introduce nuances in our results. Finally, to elucidate the clinical implications definitively, further extensive basic analysis is imperative to validate the observed protective effect of BDH1 on type 2 diabetes and delineate the specific contributions of immunocytes. Therefore, future investigations must involve the development of cellular and animal models derived from the findings of this study to further validate the protective role of BDH1 in type 2 diabetes, specifically focusing on its mediation through immune cells.

## CONCLUSION

**Fig. (1):**
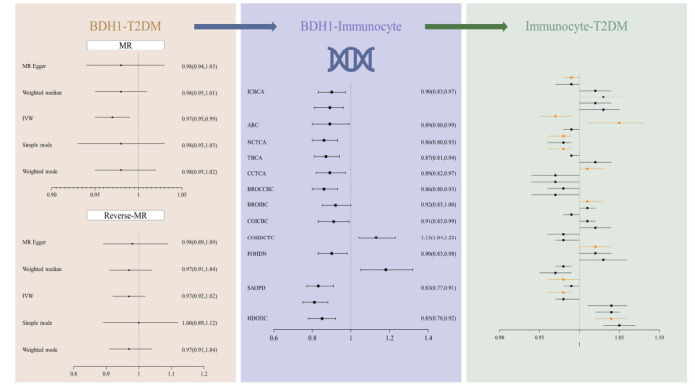
Forest plots of Mendelian randomization analysis results.

In summary, our study has revealed a causal protective effect of BDH1 on type 2 diabetes, with various immunocyte phenotypes acting as intermediaries. These findings offer novel insights into the role of the immune system in the pathogenesis of type 2 diabetes and present innovative ideas for the development of therapeutic strategies targeting BDH1 and immune pathways in the treatment of type 2 diabetes (Fig. **1**). Forest plots of Mendelian randomization analysis results.

## AUTHORS’ CONTRIBUTIONS

YYL and YYZ made equal contributions to this work by performing the statistical analysis, interpreting the results, and writing the paper, and they share the first authorship. QW proposed the idea for this study. All authors have contributed to the article and approved the submitted version.

## Figures and Tables

**Fig. (2):**
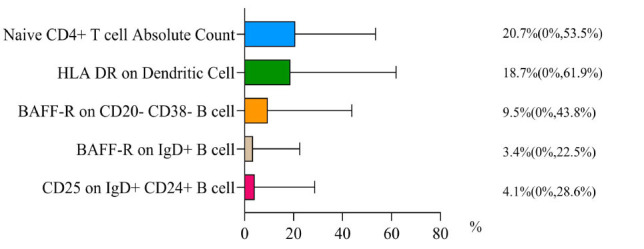
The proportion of mediators corresponding to the three immune cell phenotypes.

## Data Availability

The datasets supporting the findings of this article are included within the article.

## LIST OF ABBREVIATIONS

BDH1: β-hydroxybutyrate Dhydrogenase 1
MR: Mendelian Randomization
IVs: Instrument Variables
IVW: Inverse Variance Wighted
SNP: Single Nucleotide Polymorphisms

## STANDARDS OF REPORTING

STROBE guidelines were followed.
