# Supplementary material for: Immunocytes Play a Crucial Role as Mediators in the Protective Effects of D-β-Hydroxybutyrate Dehydrogenase 1 against Type 2 Diabetes Mellitus: A Mendelian Randomization Study
Source: Endocr Metab Immune Disord Drug Targets. 2025 May 19;26:E18715303380282. doi: 10.2174/0118715303380282250225071730 (PMC13334251; doi:10.2174/0118715303380282250225071730)
Supplement: Supplementary file 1 [file EMIDDT-26-E18715303380282_SD1.pdf]

Supplementary Material

Immunocytes Play a Crucial Role as Mediators in the Protective Effects of D-β-Hydroxybutyrate Dehydrogenase 1 against Type 2 Diabetes Mellitus: A Mendelian Randomization Study

Yi-Ying Liu[1,#] , Yue-Yang Zhang[2,3,4,5,6 ,#] and Qin Wan[2,3,4,5,6 ,*]

[1]Centre for Endocrine and Thyroid Diseases, Deyang People's Hospital, Deyang, 618000, China; [2]Department of Endocrinology and Metabolism, Affiliated Hospital of Southwest Medical University, Luzhou, 646000, China; [3]Metabolic Vascular Disease Key Laboratory of Sichuan Province, Luzhou, 646000, China; [4]Sichuan Clinical Research Center for Diabetes and Metabolism, Luzhou, 646000, China; [5]Sichuan Clinical Research Center for Nephropathy, Luzhou, China, 646000; [6]Cardiovascular and Metabolic Diseases Key Laboratory of Luzhou, Luzhou, 646000, China

Table S1. Association of the BDH1 with Immunocyte: instrumental variables used in Mendelian randomisation.

Research methodology	SNP	F-statistic	Effect allele	Other allele	Exposure			Outcome		
					β	SE	P-value	β	SE	P-value
MR	rs1050119	20.91	T	C	-0.06036	0.0132	4.81E-06	0.0143	0.0092	0.1208
	rs11708552	37.68	C	A	0.171961	0.028012	8.31E-10	-0.0339	0.0231	0.1426
	rs11709808	22.56	T	C	0.070546	0.014853	2.04E-06	-3.00E-04	0.0102	0.9792
	rs11835818	24.79	C	T	0.059379	0.011926	6.39E-07	-0.0054	0.0063	0.3855
	rs11920698	67.82	T	C	0.115555	0.014032	1.79E-16	-0.0129	0.0086	0.1366
	rs11923999	54.90	G	A	0.182772	0.024667	1.27E-13	-0.0059	0.0172	0.7339
	rs12490310	20.28	C	A	0.05397	0.011983	6.67E-06	-0.0028	0.0115	0.8092
	rs13077136	228.94	T	G	0.193794	0.012808	1.01E-51	-0.0016	0.0075	0.8345
	rs13088613	83.07	T	C	0.181255	0.019886	7.90E-20	-0.0012	0.0139	0.9304
	rs13096090	157.33	C	T	0.263022	0.020969	4.31E-36	-0.0047	0.0136	0.7279
	rs1367764	226.70	T	A	0.239188	0.015886	3.12E-51	-0.0171	0.0155	0.269
	rs147637544	69.63	G	A	0.261616	0.03135	7.13E-17	0.0088	0.0216	0.682501
	rs1835669	38.60	T	C	-0.10549	0.016979	5.20E-10	0.0168	0.0094	0.0731
	rs2484	118.97	C	T	0.226518	0.020767	1.06E-27	-0.0045	0.0103	0.6609
	rs2567350	58.10	T	C	-0.09835	0.012903	2.49E-14	0.0031	0.0073	0.6687
	rs2567358	21.86	G	T	0.066482	0.014219	2.93E-06	0.0137	0.0093	0.1391
	rs2567371	88.30	C	G	0.117801	0.012536	5.61E-21	0.001	0.0076	0.8923
	rs2686065	110.95	G	A	0.13178	0.01251	6.06E-26	-0.0033	0.0068	0.6206
	rs2686101	94.23	C	T	0.124418	0.012817	2.79E-22	-0.0131	0.0094	0.1637
	rs2686106	50.47	G	T	-0.10129	0.014257	1.21E-12	0.0112	0.014	0.4265
	rs34640900	169.11	T	C	0.245071	0.018845	1.16E-38	-0.0147	0.013	0.2585
	rs34719453	21.41	G	C	0.078655	0.017	3.72E-06	0.0109	0.0127	0.3886
	rs34942830	51.09	T	C	-0.08524	0.011925	8.80E-13	0.0029	0.0075	0.6964
	rs35414948	108.96	A	G	0.156214	0.014965	1.65E-25	6.00E-04	0.0096	0.9463
	rs35599589	43.71	T	C	0.197846	0.029926	3.81E-11	-0.0014	0.0199	0.9424
	rs35717088	46.86	A	C	-0.13879	0.020274	7.61E-12	0.0176	0.0125	0.16
	rs35902624	111.93	C	G	0.169441	0.016015	3.69E-26	-0.0052	0.011	0.636499
	rs4857528	24.85	T	C	0.073689	0.014782	6.20E-07	-0.0099	0.01	0.3233
	rs58449430	105.70	T	C	0.208242	0.020254	8.55E-25	-0.0039	0.014	0.7802
	rs58494737	29.98	G	C	0.067588	0.012343	4.36E-08	0.0096	0.0073	0.1896
	rs60524668	296.31	C	T	0.21873	0.012706	2.08E-66	-0.0018	0.0074	0.8098

	rs62282559	20.14	T	C	0.12113	0.02699	7.20E-06	-0.0295	0.0176	0.093651
	rs6782014	299.95	T	C	0.213041	0.012301	3.35E-67	-0.0052	0.0079	0.510199
	rs7616869	37.34	G	A	-0.0729	0.01193	9.93E-10	0.008	0.0075	0.2823
	rs7647263	22.72	C	T	0.058659	0.012305	1.87E-06	-1.00E-04	0.0069	0.9886
	rs77318519	20.89	C	T	-0.14768	0.032312	4.87E-06	0.0208	0.0194	0.2814
	rs79100031	21.18	A	G	0.128164	0.02785	4.19E-06	-0.0326	0.0206	0.1131
	rs7953704	22.77	G	A	-0.057	0.011945	1.82E-06	0.0016	0.0063	0.8027
	rs80015863	171.80	T	C	0.287981	0.02197	2.98E-39	-0.0036	0.0145	0.806
	rs10830963	60.12	G	C	0.0504	0.0065	1.20E-14	-0.03356	0.013066	0.010205
	rs10965250	507.17	A	G	-0.1644	0.0073	#######	0.004606	0.015822	0.771051
	rs11257655	246.81	T	C	0.1084	0.0069	2.36E-55	-0.00064	0.014637	0.964904
	rs1128249	101.54	T	G	-0.0786	0.0078	9.30E-24	-0.00523	0.012198	0.667924
	rs11514706	91.01	C	A	0.0601	0.0063	8.55E-22	0.004046	0.011952	0.734911
	rs11651052	196.00	G	A	-0.091	0.0065	5.35E-44	0.020769	0.011957	0.082387
	rs1215468	117.83	G	A	-0.0749	0.0069	3.91E-27	-0.02273	0.013363	0.088867
	rs12546365	34.36	C	T	-0.0381	0.0065	4.66E-09	-0.00233	0.011966	0.845706
	rs1260326	123.07	C	T	0.071	0.0064	6.44E-29	0.032852	0.012206	0.007111
	rs12698897	68.12	A	T	0.0586	0.0071	1.23E-16	0.000177	0.013943	0.989947
	rs13266634	262.44	T	C	-0.1053	0.0065	1.97E-59	0.001368	0.012946	0.9159
	rs139722172	50.14	G	C	-0.0786	0.0111	1.82E-12	0.009988	0.015383	0.51621
	rs1515104	93.44	A	T	0.0754	0.0078	6.57E-22	-0.01782	0.012395	0.150548
	rs1574285	106.45	T	G	-0.065	0.0063	1.17E-24	-0.0045	0.012016	0.708256
	rs17036160	95.06	T	C	-0.1092	0.0112	1.73E-22	-0.01903	0.017496	0.276772
	rs1977832	295.84	T	G	-0.1118	0.0065	2.73E-65	0.003348	0.01207	0.781626
	rs2237897	885.39	T	C	-0.2678	0.009	#######	0.009493	0.02862	0.740191
	rs2583938	80.77	A	T	0.0692	0.0077	2.96E-19	0.002833	0.015959	0.859194
	rs28375915	41.83	A	G	-0.0498	0.0077	1.21E-10	0.002587	0.011917	0.828053
	rs340874	72.11	C	T	0.0535	0.0063	3.51E-17	-0.00203	0.011918	0.864696
	rs34872471	1066.63	C	T	0.2972	0.0091	#######	-0.01704	0.013088	0.19278
Re-MR	rs35385487	72.92	G	A	-0.076	0.0089	1.47E-17	0.024424	0.032397	0.450969
	rs35473599	35.36	A	G	0.0553	0.0093	2.67E-09	-0.01307	0.013848	0.345272
	rs4273712	76.43	G	A	0.0577	0.0066	2.98E-18	-0.01258	0.013434	0.348964
	rs429358	64.52	C	T	-0.0739	0.0092	1.02E-15	0.020846	0.017373	0.230217
	rs4711750	50.57	A	T	0.0448	0.0063	1.22E-12	0.00472	0.01191	0.69181
	rs4731701	55.44	T	C	-0.0484	0.0065	1.31E-13	-0.0088	0.011924	0.460393
	rs4854343	57.16	A	G	0.0688	0.0091	5.22E-14	0.002236	0.015938	0.888344
	rs5215	94.76	T	C	-0.0623	0.0064	2.41E-22	-0.0241	0.012298	0.050066
	rs554833	47.91	T	C	0.0443	0.0064	4.19E-12	0.01826	0.012359	0.139622
	rs56094641	285.56	G	A	0.1166	0.0069	2.23E-64	-0.00042	0.012156	0.972558
	rs633715	42.86	C	T	0.0491	0.0075	5.66E-11	-0.01644	0.0152	0.279431
	rs6567160	106.69	C	T	0.0785	0.0076	4.88E-25	0.017206	0.013972	0.218174
	rs6813195	88.25	T	C	-0.062	0.0066	4.38E-21	0.000545	0.013141	0.966977
	rs7132908	35.64	A	G	0.0394	0.0066	2.63E-09	-0.01881	0.012159	0.121767
	rs7172432	91.92	G	A	-0.0604	0.0063	8.28E-22	0.029885	0.011999	0.012753
	rs7250869	56.37	C	T	-0.0488	0.0065	5.17E-14	0.016686	0.012372	0.177401
	rs72802365	64.00	C	G	-0.1168	0.0146	1.21E-15	-0.01816	0.020818	0.383099
	rs72964564	93.20	C	A	-0.1004	0.0104	3.96E-22	-0.00524	0.0144	0.715708
	rs755249	64.21	T	C	0.0617	0.0077	1.45E-15	-0.027	0.013925	0.052501
	rs8043085	45.17	T	G	0.0457	0.0068	1.84E-11	-0.00158	0.013939	0.909872
	rs860262	96.50	A	C	-0.0668	0.0068	9.18E-23	0.007194	0.011934	0.546509
	rs9368222	552.25	A	C	0.1551	0.0066	#######	0.00096	0.013247	0.942284
